# Unnecessity of routine chest tube drainage after patent ductus arteriosus ligation in preterm neonates

**DOI:** 10.1186/s13052-023-01548-y

**Published:** 2023-10-20

**Authors:** Youngok Lee, Hanna Jung

**Affiliations:** Department of Thoracic and Cardiovascular Surgery, Kyungpook National University Hospital, Kyungpook National University School of Medicine, 130 Dongdeok-ro, Jung-gu, Daegu, 41944 Republic of Korea

**Keywords:** Drainage, Intensive care unit, Neonate, Patent ductus arteriosus, Preterm birth, Thoracotomy

## Abstract

**Background:**

Conventionally, a chest tube drainage is placed following patent ductus arteriosus (PDA) ligation to monitor possible bleeding and drain air or effusion postoperatively. However, the necessity of chest tube drainage after thoracotomy in PDA ligation is controversial. We evaluated the feasibility and safety of omitting chest tube drainage in preterm neonates who underwent PDA ligation via thoracotomy.

**Methods:**

We retrospectively reviewed the medical records of 56 preterm neonates who underwent surgical ligation of PDA via thoracotomy in the neonatal intensive care unit between January 2014 and March 2022.

**Results:**

The median gestational age was 26.9 (interquartile range [IQR]: 25.9–28.8) weeks and the median body weight at birth was 895 (IQR: 795–1190) g. The median age on the day of surgery was 17.0 (IQR: 10.0–22.0) days and the median body weight on the day of surgery was 1100 (IQR: 958–1410) g. The median operative time was 44.5 (IQR: 35.5–54.0) minutes. There were no intraoperative events or procedure-related deaths. On postoperative chest radiographs, no patients had major complications, such as pneumothorax or hemothorax. Nineteen patients (34%) had minor complications of subcutaneous emphysema around the thoracotomy site. No patients required additional chest tube drainage for postoperative bleeding, pleural effusion, or progressive subcutaneous emphysema. No patients had surgical wound infections. There were seven in-hospital mortalities, which were unrelated to the surgery.

**Conclusions:**

Omitting chest tube drainage is feasible and safe for the postoperative management of preterm neonates undergoing PDA ligation via thoracotomy.

## Background

Patent ductus arteriosus (PDA) is one of the most common congenital cardiac diseases in preterm neonates. Although there are numerous studies regarding the treatment of preterm PDA, which is a highly debated discussion among cardiologists, surgeons, and neonatologists, it is indisputable that certain preterm neonates with PDA require surgical ligation because of the possibility of uncontrolled congestive heart failure [1–3].

Conventionally, a chest tube drainage is placed inside the pleural cavity following PDA ligation to allow natural drainage of excess air or effusion, or to detect surgical bleeding during the postoperative period. However, based on our experience, the majority of neonates did not exhibit surgical bleeding, air leakage, or pleural effusion postoperatively. Therefore, we initially examined the possibility of removing the chest tube on the morning of the day after the surgery. Further, many studies related to thoracoscopic lung surgery were found to support early removal or even omitting chest tube drainage after wedge resection of the lung, substantiating the feasibility of our strategy [4–9]. The absence of surgical manipulation of the lung and the minuscule lung injury during PDA ligation via thoracotomy makes chest tube drainage unnecessary.

Starting from 2014, preterm neonates have not undergone chest tube drainage after PDA ligation. We evaluated operative outcomes in conjunction with the process of recovery of neonates after the surgery. Based on these observations, this study assessed the practicality and safety of omitting chest tube drainage after PDA ligation in preterm neonates.

## Methods

### Study population

The institutional review board of Kyungpook National University Hospital approved this study. We retrospectively reviewed the medical records of 56 preterm neonates who underwent surgical ligation of PDA via thoracotomy in the neonatal intensive care unit between January 2014 and March 2022.

### Preoperative considerations

All surgeries were performed in the neonatal intensive care unit to avoid mishaps that could occur during the transport of neonates to the operating room. All patients were under ventilator support and the optimal ventilator settings were managed by a neonatologist. Subsequently, visual confirmation of the endotracheal tube position and bilateral lung conditions were performed using daily routine chest radiographs. These assessments were important to prevent the endotracheal tube from falling out or one-lung ventilation during surgery, as the patient’s head was visually unobservable after the body was draped completely. We ensured that two peripheral intravenous accesses with extension lines were installed away from the surgical field. Heart rate was monitored using a three-lead continuous electrocardiogram, and pulse oxygen saturation of the right hand (preductal) and foot (postductal) was measured using pulse oximetry. Noninvasive blood pressure was checked using an appropriately sized blood pressure cuff regularly. The anesthetic agents and inotropic agents were managed and closed monitored by a neonatologist during the entire surgery time [10].

### Surgical techniques

Under general anesthesia, the patient was positioned with the left side up with the left arm supporting over the head to elevate the left scapula. Careful retraction of the intercostal space for exposure of the pleural cavity was performed through the left posterolateral thoracotomy at the third intercostal space. The left lung was then gently retracted anteriorly. The mediastinal pleura were dissected and opened above and below the aortic end of the ductus. The vagus and left recurrent laryngeal nerves were visually confirmed to aid in clear and unobstructed views of the ductus [11]. The PDA was ligated using a single titanium hemostatic clip (SLS-CLIP® VITALITEC, Peter Surgical, Boulogne-Billancourt, France) or by the double-ligation transfixion technique (using 4−0 black silk and 6−0 Surgipro sutures). After PDA ligation, the mediastinal pleura was closed using 7−0 Prolene sutures. Before the chest wall closure, a 5-Fr silicone feeding tube was placed into the pleural cavity. When closing the chest wall, the end of the feeding tube was placed in an underwater seal to evacuate the air within the pleural space; the patient was kept on mechanical ventilation during the process. The feeding tube was removed from the pleural cavity once air leakage ceased [4, 7, 12]. Subsequently, the subcutaneous layer and the skin closure were completed.

### Classification of subcutaneous emphysema

**Fig. 1:**
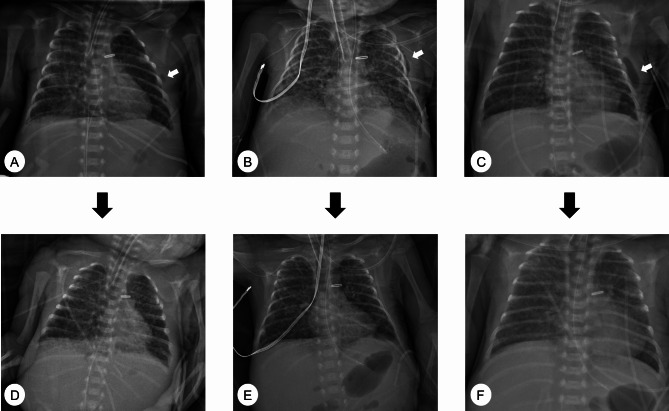
Chest radiographs showing subcutaneous emphysema (**A-C**, white arrow) around the thoracotomy site with full expansion of the left lung. The severity of subcutaneous emphysema was graded as scanty (**A**), mild (**B**), or moderate (**C**). All 19 patients’ subcutaneous emphysema was spontaneously absorbed (**D-F**) on the follow-up chest radiographs without active intervention

The diagnosis of the subcutaneous emphysema is confirmed by the detection and presence of air inside the soft tissue by the postoperative chest radiography [13]. We classified the severity of subcutaneous emphysema in four grades. Air distribution limited to one intercostal space was classified as scanty (Fig. 1A). Air excess more than one intercostal space and linear shape was classified as mild (Fig. 1B). Air excess more than one intercostal space and oval shape (diameter longer than one intercostal space) was classified as moderate (Fig. 1C). Air distribution up to neck area was classified as severe.

### Statistical analysis

Continuous variables are expressed as medians and interquartile ranges for non-normally distributed data. Categorical variables are expressed as numbers and percentages. All statistical analyses were performed using R (version 4.0; R Foundation for Statistical Computing, Vienna, Austria, http://www.R-project.org).

## Results

The patients’ demographic characteristics are presented in Table 1. Of the 56 patients, the median gestational age was 26.9 (interquartile range [IQR]: 25.9–28.8) weeks, and 36 patients (64.3%) were extremely preterm neonates. The median birth body weight was 895 (IQR: 795–1190) g, and 35 patients (62.5%) were extremely low birth weight neonates. The median age on the day of surgery was 17.0 (IQR: 10.0–22.0) days, and median body weight on the day of surgery was 1100 (IQR: 958–1410) g.

**Table 1:** Characteristics of the patients (n = 56)

Characteristic	Value
Sex	
Female	29 (51.8%)
Male	27 (48.2%)
Gestational age (weeks)	26.6 (25.9–28.8)
Extremely preterm neonates (< 28 weeks, n)	36 (64.3%)
Birth body weight (g)	895 (795–1190)
Extremely low birth weight neonates (< 1000 g, n)	35 (62.5%)
Age on the day of surgery (day)	17.0 (10.0–22.0)
Body weight on the day of surgery (g)	1100 (958–1410)

Values are presented as medians (interquartile ranges) or numbers (%)

The median operative time was 44.5 (IQR: 35.5–54.0) minutes. Most patients (54 patients, 96.4%) underwent PDA ligation using a single titanium hemostatic clip. Two patients underwent PDA ligation by double ligation transfixion technique, because we did not have properly sized clips. Blood loss was minimal in all patients (a small unmeasurable quantity) and there were no intraoperative complications.

Immediate postoperative chest radiography confirmed the full expansion of the left lung. Subcutaneous emphysema developed around the thoracotomy site in 19 (34%) of 56 patients. Among 19 patients with subcutaneous emphysema, 9 patients had scanty, 6 had mild, and 4 had moderate subcutaneous emphysema. In all, 18 of 19 patients’ subcutaneous emphysema resolved completely within two days after surgery in the follow-up chest radiographs without any active intervention (Fig. 1D-F). One patient with moderate subcutaneous emphysema spontaneously absorbed air within four days after surgery. No patients required additional chest tube drainage for postoperative bleeding, pleural effusion, or progressive subcutaneous emphysema. No patients had surgical wound infections.

There were no operation related deaths. Postoperative follow-up echocardiography results were reviewed on 45 of 49 living patients, and only one patient had a residual PDA shunt. Four patients, who had no postoperative follow-up echocardiographic records, had no audible heart murmur representing residual PDA shunt. The median follow-up duration at the outpatient clinic was 23.5 (IQR: 8.0–34.5) months for 48 of 49 living patients.

There were seven in-hospital mortalities, which were unrelated to the surgery. All seven patients were extremely low birth weight and extremely preterm neonates. Four patients underwent PDA ligation within two weeks after birth and died within 30 days postoperatively. Of them, one patient died from intraventricular hemorrhage aggravation from grade II to grade IV and other three patients died of panperitonitis. The remaining three of seven patients underwent PDA ligation two weeks after birth and died over 30 days postoperatively. One patient died of small bowel perforation and two patients died of bronchopulmonary dysplasia.

## Discussion

Conventionally, a chest tube drainage is placed following PDA ligation to monitor possible bleeding and drain air or effusion postoperatively. The chest tube drain is typically inserted even in the absence of lung injury or bleeding. In most cases, after PDA ligation via thoracotomy, a chest tube drain is inserted for the evacuation of possible trapped air inside the thoracic cavity after skin closure and not for the drain of air leakage from potential lung injury as a surgical complication. Therefore, most patients were not found to have tangible pleural effusion or air leakage after PDA ligation. Instead, chest tube drainage is associated with significant thoracotomy pain, risk of infection, excessive pleural reactions, or impaired pulmonary function [9, 14]. Particularly, owing to their prematurity, preterm neonates are known to have many factors that predispose them to an increased risk of infection or sepsis [15]. Moreover, preterm neonates’ lung can be easily wounded by a relatively rigid chest tube because the lung parenchyma is very fragile.

In our experience, we evacuate the air by water-sealing with a 5-Fr silicon feeding tube during chest wall closure and fully expand the left lung. Neonates with chest tube drainage placed after PDA ligation had no air leak or effusion drained during the 24 h after surgery. One of the major concerns for doctors was the management of chest bottles. As there were no customized closed water-sealed chest bottles suitable for 5-Fr silicon feeding tubes, we assembled an open water-sealed chest bottle, which was unsanitary and easily spillable, causing the possibility of infection in the neonates. We always considered the necessity of a chest tube drainage, and a few studies indicated no risk of complications without routine chest tube drainage after PDA ligation [12, 16–18]. Moreover, previous reports have addressed the feasibility and safety of omitting chest tube drainage after thoracoscopic lung surgeries [4–9]. Therefore, we began omitting chest tube drainage in selected cases from 2013; since 2014, our institution has not routinely employed chest tube drainage in the pleural cavity after PDA surgical ligation in preterm neonates. In our study, no patients had significant postoperative complications, such as pneumothorax or hemothorax. However, 19 patients had minor complications of subcutaneous emphysema around the thoracotomy site. No patients required additional chest tube drainage for postoperative bleeding, pleural effusion, or progressive subcutaneous emphysema.

We also reviewed the mortality cases. As mentioned in the Results, all seven patients were extremely low birth weight and extremely preterm neonates. Four of the seven patients who underwent PDA ligation within two weeks preoperatively had severe generalized edema due to congestive heart failure, multi-organ dysfunction, and septic shock, barely maintained vital signs, and required careful consideration for PDA ligation. Neonatologists and pediatric cardiologists felt that PDA ligation might relieve congestive heart failure and eventually help overcome the sepsis state; however, unfortunately, the patients died within 30 days postoperatively.

Currently, we do not employ a chest tube drain in the pleural cavity after PDA ligation even in full-term born neonates. As previously mentioned, uncomplicated PDA ligation is a relatively short-time procedure with minimal or no damage to intrathoracic structures, causes no blood loss, does not involve lung resection, and the pleura absorbs a small amount of inflammatory pleural effusion or blood [12, 16]. Therefore, we also found no fluid and air with or without a chest tube drainage in the pleural cavity, corroborating the unnecessity of routine chest tube drainage after PDA ligation in preterm neonates.

Nevertheless, there are also several limitations to our study. Firstly, it is a single-center retrospective study with a small sample size, and the experimental results need to be further confirmed by a larger sample size study. Secondly, we had to use a non-commercialized product (5-Fr feeding tube with assembled open water-sealed chest bottle) since the patients’ pleural cavity was relatively too small for a 10-Fr trocar catheter or 16-Fr chest tube. Finally, we did not use the lung ultrasound to check for air leak, pneumothorax, or effusion. However, lung ultrasound would be a good option instead of chest radiographs as it reduces patients’ exposure to X-rays.

## Conclusions

Omitting chest tube drainage is feasible and safe for the postoperative management of preterm neonates undergoing PDA ligation via thoracotomy. A minor complication was subcutaneous emphysema around the thoracotomy site, spontaneously absorbed without active intervention.

## Data Availability

The datasets used and/or analysed during the current study available from the corresponding author on reasonable request.

## Abbreviations

PDA: Patent ductus arteriosus
IQR: Interquartile range
